# The Assessment of Quality of Products Called Sandalwood Oil Based on the Information Provided by Manufacturer of the Oil on Polish, German, and English Websites

**DOI:** 10.1155/2021/9934143

**Published:** 2021-07-12

**Authors:** Magdalena Hartman-Petrycka, Agata Lebiedowska

**Affiliations:** Department of Basic Biomedical Science, Faculty of Pharmaceutical Sciences in Sosnowiec, Medical University of Silesia, Kasztanowa 3, Sosnowiec 41-205, Katowice, Poland

## Abstract

**Background:**

Sandalwood oil is one of the most valuable raw materials worldwide. As a highly valued product, it has its own regulations based on the ISO 3518 standard, which clearly informs producers, distributors, and consumers of the requirements to be met. The aim of this study was to assess the quality of products called sandalwood oil based on the information provided by the manufacturer of the oils on Polish, German, and English websites.

**Methods:**

A Google search was utilized to collect data on sandalwood oil offered by producers and distributors in Polish and foreign markets. Information from 50 websites in each of the aforementioned languages, including the description of sandalwood oil properties on websites, method for using it, safety limitations, and presence of a product description consistent with the INCI recommendations, was gathered using Microsoft Excel software and was analyzed. The information that enabled us to estimate the quality of the oils was the botanical name of the oil-bearing plant and the price. Good-quality oils were considered to be oils with the botanical name *Santalum album* in the description and with a price not considerably less than the price of white sandalwood oils sold by reliable distributors who control the quality of the oils by chromatography. Ultimately, the lower price limit for one milliliter of the oil was established as PLN 21. *Results and Conclusions.* Good-quality sandalwood oils derived from the *Santalum album* plant at a price equal to or greater than the chromatographically tested items amounted to a negligible percentage of products sold online. Without knowing the botanical name of the essential oil plant and the price range of unadulterated sandalwood oil, the likelihood of buying a reliable product is low on all of the analyzed websites, with the lowest probability being observed on the Polish websites.

## 1. Background

The lockdown related to the COVID-19 pandemic has spread across the globe. As people have been confined to their homes, they have transferred their lives to the Internet, and many new instructional videos about performing beauty treatments and making natural cosmetics have appeared. The necessary products have been purchased mainly via the Internet. Although the restrictions related to the COVID-19 pandemic have decreased, interest in online shopping continues to grow [1].

Sandalwood oil is one of the most valuable raw materials and is widely used in cosmetics and natural medicine, showing a number of positive effects on the skin and the entire body. The essential oil obtained from East Indian sandalwood, also known as sandalwood (*Santalum album* L.) [2, 3], is considered to be authentic sandalwood oil. The sandalwood is one of the most valuable trees in the world [3]. The raw materials from this tree were used thousands of years ago in India [2], where it can still be found in the wild, as well as in Indonesia, China, and the Malay Archipelago [4].

Essential oils are substances derived from plants that are under many different legal regulations ensuring their appropriate quality, such as the ISO 9235 standard (Aromatic natural raw materials—vocabulary). ISO standards specify the method for obtaining oils, the need to use the term “essential” in the name of the product, and the requirement to inform consumers about the country of origin of raw materials [5]. Sandalwood oil, as a highly valued product, has its own regulations based on the ISO 3518 standard, which clearly informs producers, distributors, and consumers concerning the requirements to be met [6]. Sandalwood oil is produced in the process of distillation only of the scleroderma and the roots of *Santalum album* L. Sandalwood oil is a transparent raw material that is slightly viscous, slightly yellow, and almost colorless. This material is characterized by a persistent, woody, and sweet fragrance, which is described as “oriental.” An important property described in ISO 3518 is the content of free alcohols, which should reach 90% (*Z*-*α*-santalol from 41 to 55% and *Z*-*β*-santalol from 16 to 24%) [2, 6]. The high value of sandalwood oil in aromatherapy is additionally achieved by the content of more than 125 ingredients, including other forms of santalol: bergamotol, lanceol, and santalenes [7, 8].

Sandalwood oil has a special value in perfumery, and its use guarantees not only an oriental, woody scent but also enhances and preserves the aromatic properties of other fragrance ingredients. It is popular to combine this oil with floral fragrance note oils, such as rose or lavender [3, 9]. Sandalwood oil is expensive; in Poland, 1 mL costs more than 20 PLNs; for comparison, citrus essential oils cost approximately 1.2 PLN for 1 mL. The high price is related to the difficulty of obtaining this material caused by previous excessive exploitation, not the lack of raw material efficiency [9, 10]. The price prompts many perfume and cosmetic manufacturers to look for synthetic or natural substitutes for sandalwood oil. The original, despite its high value, remains an irreplaceable ingredient in many noble fragrance compositions [3, 9].

In traditional medicine, which dates back to the heyday of Ayurveda and ancient China, sandalwood oil has been used as an antidepressant, sedative, and antiseptic [3, 4, 7, 9]. In natural medicine, it is recommended to use sandalwood oil for colds and inflammation of the mouth, bronchi, or urinary tract [3].

Modern medicine has confirmed the ability of sandalwood oil to inhibit inflammatory reactions and deactivate free radicals. Sandalwood oil is an inhibitor of 5-lipoxygenase, and it also reduces the expression of interleukin 1b in keratinocytes [7]. Sandalwood oil has an antibacterial effect and develops antibiotic resistance, for example, in *Staphylococcus aureus* resistant to methicillin [7]. From a dermatology perspective, the effect of sandalwood oil against bacteria, such as *Propionibacterium acnes, Staphylococcus epidermidis* or *Streptococcus pyogenes*, which are involved in the etiopathogenesis of many skin diseases, is very important [6, 11–13]. Its effectiveness against fungi such as Microsporum, Trichophyton, and Epidermophyton, as well as yeasts of the genus *Candida*, has also been shown, enabling its use in the treatment of mycoses and yeasts. Sandalwood oil is also active against the herpes simplex viruses HSV 1 and HSV 2 [7]. In dermatology, sandalwood oil is also used in psoriasis or atopic dermatitis treatments [14, 15]. Studies exploring the effect of sandalwood oil inhalation on hay fever have confirmed its positive reaction with the nasal mucosa and the reduction of the severity of allergies [16]. Synthetic ingredients, analogous to those contained in sandalwood oil called “sandaroles,” promote wound regeneration and reduce the visibility of scars. Long-term exposure to this substance has a positive effect on the proliferation and migration of keratinocytes, which are favorable to renewal of the epidermis [17].

Numerous studies have indicated the anticarcinogenic effects of sandalwood oil or its main components on urinary bladder, oral cavity and prostate cancers [7]. The ability to induce autophagy and apoptosis in keratinocytes indicates their possible action in preventing the formation of precancerous skin conditions, as well as their transformation into skin cancers [7]. The activation of early antigens against Epstein–Barr virus is also considered to be an anticancer effect of the oil [18]. The anticancer effect of sandalwood oil or its components in breast cancer has been reported, both in animal studies in which the number and size of tumors decreased [19] and in MCF-7 and MCF-10A breast cancer cell lines in which the double strands of DNA were broken [20]. In clinical trials, a cream containing sandalwood oil and turmeric has been shown to reduce the formation of radiation dermatitis in women exposed to radiation as part of breast cancer treatment [21].

The use of sandalwood oil in aromatherapy to relieve stress, symptoms of depression, and insomnia is popular [22]. Studies conducted in mice have shown the calming effect of *α*-santalol on the nervous system through pharmacological transfer evidenced by the flow of this component to the brain [23]. The antidepressant effect of sandalwood oil is related to the direct stimulation of the pineal gland after inhalation, resulting in the release of serotonin [24]. The influence of santalol on insomnia and shortening the time to wake up suggests the possibility of using sandalwood oil for inhalation in elderly individuals with dementia [25].

Consuming sandalwood oil is not a popular method of using it. However, this material is used in food production, in which it mainly serves as an ingredient that gives food the desired taste, and it is regulated by the Food and Drug Administration (FDA) and the European Council [3]. Animal studies have shown that the oral administration of sandalwood oil lowers free blood glucose and levels of total cholesterol, LDL, and triglycerides and shows a supportive effect in the treatment of systemic diseases, such as diabetes [26].

The widely described properties of sandalwood oil occur only in products with an appropriate quality composition. The average consumer cannot verify it by, for example, using chromatography and therefore relies only on the information provided by the manufacturer, which can be found on packaging or the distributor's website.

## 2. Objective

The aim of the study was to assess the quality of products called sandalwood oil based on the information provided by the manufacturer of the oil on Polish, German, and English websites. Complementary goals of the study include the investigation of descriptions of sandalwood oil properties, the methods of its use and safety limitations, and the analysis of product description consistent with the recommendations of the International Nomenclature of Cosmetic Ingredients (INCI).

## 3. Methods

A Google search was used to collect data on sandalwood oil offered by producers and distributors on the Polish and foreign markets. The search settings were adjusted depending on the phrase used; that is, when analyzing the Polish-language websites, the search tools were set to “Poland”; on the German-speaking market, they were set to “Germany”; and on the English-speaking market, they were set to “United States” to obtain the most reliable results.

The key phrases that were used to gather information for analysis were “olejek sandałowy” (Polish), “sandelholzöl” (German), and “sandalwood oil” (English). Information from 50 websites in each of the aforementioned languages was analyzed. The content of websites was analyzed according to the search order, excluding positioned pages.

Only the search results that led directly to the website of a product offered by the distributor and not to the home page of the store or company were analyzed. Only the products that had a price specified and might have been purchased were considered. If the product was offered in several different volumes/weights, the smallest available version of the product size was considered.

Information on the description of sandalwood oil properties on websites, method for using it, safety limitations, and presence of a product description consistent with the INCI recommendations was gathered using Microsoft Excel software. The information that enabled us to estimate the quality of the oils was the botanical name of the oil-bearing plant and the price. Good-quality oils were considered to be oils with the botanical name *Santalum album* in the description and with a price not considerably less than the price of white sandalwood oils sold by reliable distributors, who control the quality of the oils by chromatography. Ultimately, the lower price limit for one milliliter of oil was established as PLN 21.

## 4. Results

The largest percentage of websites that provided a description of sandalwood oil properties were in English (98%), while Polish websites provided such information in 96% of cases. The lowest percentage of the sandalwood oil property description, presented only on 66% pages, was provided by the German market.

Fifty-six percent of English websites mentioned certain safety rules when using sandalwood oil. On Polish websites, the results considering the same issue decreased to 38%. The smallest percentage of pages with information about the safety limitations of sandalwood oil was recorded for the German market (18%).

On the Polish websites, the full product composition consistent with the INCI recommendations was presented on 46% of websites; on the English market, it was presented on 28% of websites, and on the German market, it was presented on only 14% of websites.

**Figure 1:**
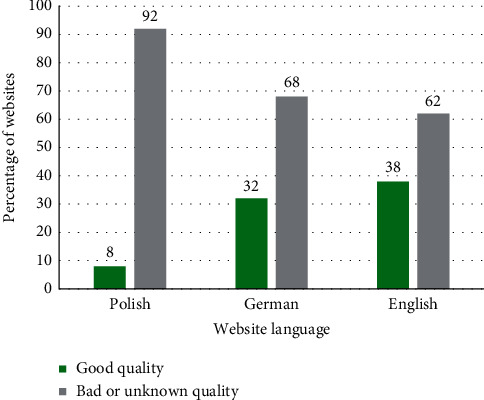
Percentage of good-quality oils available on websites in Polish, German, and English.

According to the criteria established for this study, essential oils can be considered of a good quality only for 8% of the products on Polish websites compared with 32% for German websites and 38% for English websites (Figure 1).

**Figure 2:**
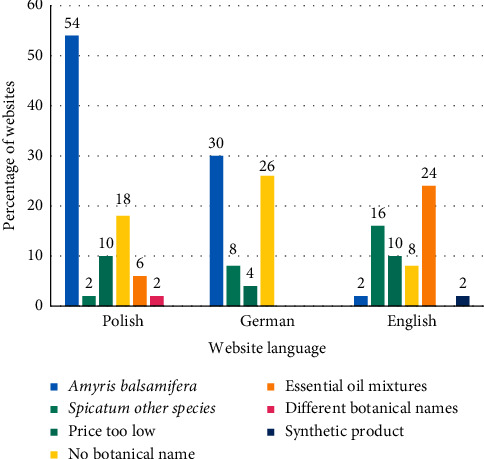
Reasons for qualifying the products as bad-quality oils.

Among oil-bearing plants other than *Santalum album*, from which oil under the name of sandalwood oil is obtained, West Indian sandalwood *Amyris balsamifera* is dominant, especially on the Polish market, constituting 54% of the products (Figure 2). The widespread presence of this material is also notable on German websites (30%). The English online market is different; *Amyris balsamifera* oils were sold on only 2% of websites, but there are oils from other sandalwood species: *Santalum spicatum* (10%), *Santalum australedonicum* (2%), and *Santalum paniculatum* (4%) (Figure 2).

The smallest percentage of products with the botanical name *Santalum album* included in the description, the price of which was too low, considering the cost of producing sandalwood oil, is notable on the websites in German (4%). Ten percent of this type of product was found on both Polish and English websites.

The average price for 1 mL of sandalwood oils under the botanical name *Santalum album*, which can be considered reliable according to the criteria for this study, was the lowest on websites in Polish at PLN 28.19 and the highest on websites in English at PLN 50.79. On websites in German, the average price per milliliter of oil was PLN 45.35. A similar dependence is also notable for products that cost less than PLN 21/mL, even though the botanical name *Santalum album* is declared by the producers. Among these products, the average price is also significantly higher for oils sold on English websites, that is, PLN 11.25/mL, compared with the Polish and German online markets, where the values are comparable: PLN 7.36/mL and PLN 7.60/mL, respectively.

A frequent reason for not considering a given product as good-quality sandalwood oil is the lack of a botanical name on the distributor's website, which is especially notable on German websites (26% of products).

Blends of oils in vegetable oil with the described composition are most often observed on English-language websites (10%), and a product described as synthetic oil was also observed there. One Polish website was selling oil, the description of which contained contradictory information regarding the oil-bearing plant. In the photo of the oil packaging, there was a different botanical name than in the text describing the oil properties. Well-described mixtures appeared on 6% of Polish websites.

The purchase of oil from a given website is affected by the position of the website. On websites in Polish, the first three oils meeting the quality criteria were in the 10th, 12th, and 14th positions, respectively, on the list of searched websites; on the German websites, the first three good-quality oils were in the 1st, 3rd, and 4th positions, respectively; on English websites, they were in the 2nd, 6th, and 7th positions, respectively (Table 1).

## 5. Discussion

Sandalwood oil is widely used and can be used in natural medicine, especially in aromatherapy. Moreover, modern science has demonstrated the benefits of its application in dermatology, cosmetology, and even oncology [27]. Sandalwood oil can be used by professionals but also commonly, without specialist preparation, for example, in saunas or for air aromatization. Essential oil sellers should provide a good-quality product and basic information for safe use. However, sandalwood oil sellers have not lived up to the challenge in regard to the quality and product description. Each of the analyzed online markets had serious shortcomings, although the authors evaluated the Polish market as the worst with only 8% trustworthy products and the English market as the best (38% trustworthy products).

Websites that distribute substances called “sandalwood oil,” mostly English and Polish, describe the oils' properties. In the German-speaking market, this description was observed considerably less frequently and covered only 66% of pages. This study did not undertake a detailed assessment of the information about sandalwood oil on websites, but a cursory analysis indicated that most of them described antidepressant, sedative, and antiseptic properties, which have been known in natural medicine for centuries [3, 4, 7, 9]. This phenomenon is undoubtedly beneficial; it allows us to know the richness of the oil for those who are recently acquainted with aromatherapy. Such a description can also inspire deeper knowledge and result in more reliable studies than essential oil seller websites.

The use of essential oils is associated with the risk of allergic reactions, irritation, or photosensitization, especially if they are used incorrectly, for example, in undiluted form [10]. Despite the relatively strong effect of sandalwood oil, which is related to its rich composition, only a small proportion of Polish and German websites contained a safety rule description of the use of this product. Most pages did not even mention the necessity of diluting the essential oil, which is a basic principle of aromatherapy [28]. The assumed criterion, the specification of safety limitations for sandalwood oil use, was met to the greatest extent by English websites (56%), suggesting that English distributors are most aware of the risks associated with inappropriate use of this oil. This dependency might also be related to stricter laws in English-speaking countries, especially the United States, in terms of the producer's responsibility for the safety of its product.

All cosmetic products, which also include sandalwood oil, should have a description consistent with the International Nomenclature of Cosmetics Ingredients (INCI) guidelines. According to these guidelines, the chemical composition of each product should be described in English. The description should start with the ingredient found in the largest amount in the product. If the product is of plant origin, the Latin botanical name of the plant must be provided [29]. The website analysis showed that the sandalwood oil distributors did not undertake the necessary effort to properly describe the product. The full description compatible with the INCI guidelines appeared only on 45% of Polish and 14% of German websites. Perhaps the leaflets attached to the product indicated the full composition in accordance with the INCI guidelines, but distributors, while emphasizing advertising, did not devote proper attention to this aspect, which could contribute to increasing disinformation on the aromatherapy market, both in Poland and abroad.

When examining the differences in the descriptions of the products depending on the language of the website, the historical and cultural aspects in Polish-, German-, and English-speaking countries are worth mentioning. The history of aromatherapy development in Poland is shorter than that in our Western neighbors [30–32]. Aromatherapy has developed differently over the years in Poland and Western countries. The use of essential oils, which is now called aromatherapy, was introduced to modern Europe by the French chemist Rene Gattefosse in 1920 due to the use of lavender oil to help heal wounds. In the literature, this method was described by this researcher for the first time in a book published in 1937. Another French scientist, Dr. Jean Valnet, whose book was published in 1964, is responsible for the development of aromatherapy in Germany. Essential oils also gained popularity quite early in the United Kingdom, which had colonies in India, the homeland of sandalwood oil. The United Kingdom, due to notable participation in exports, also contributed to the spread of aromatherapy in the United States and Australia [30, 33]. In Poland, the real development of aromatherapy was initiated by Pollena-Aroma, along with the potpourri product introduction in 1989. The most important event for aromatherapy in Poland was the publication of the first textbook in Polish on the use of essential oils, *Pachnąca Apteka–Tajemnice Aromaterapii*, in 1992, 55 years after Rene Gattefosse's publication [31].

Notably, medicine and natural cosmetics are an increasingly popular subject for the average consumer [34], and this popularity does not always go hand in hand with thorough knowledge. A consumer buying a product called sandalwood oil does not always have an idea that he or she might be manipulated and, at the same time, does not have the information that enables him or her to distinguish between genuine and adulterated essential oil. For example, the lack of awareness from which plant sandalwood oil should be obtained creates the risk of unknowingly using products that do not offer the properties expected by the consumer, while a very common trick of essential oil sellers is selling oil from a plant other than *Santalum album*. In Poland, the situation can be considered scandalous in this aspect. Every second essential oil sold via Polish websites under the name “sandalwood oil” actually comes from the *Amyris balsamifera* plant, belonging to the *Rutaceae* (citrus) family. On German websites, the situation is only slightly better; 30% of websites offered oil from the same plant. *Amyris balsamifera* is a small tree growing in Haiti and Jamaica [2]. Although this plant is called West Indian sandalwood in Polish, botanically, it has nothing in common with the sandalwood family. The oil obtained from the *Amyris balsamifera* plant differs in chemical composition from sandalwood oil and thus in its effect on the body. The oil from the *Amyris balsamifera* plant has become a very popular and less expensive alternative to actual sandalwood oil due to its characteristic woody fragrance, similar to that of sandalwood oil. However, this material cannot be used as a substitute for sandalwood oil in aromatherapy [2]. According to the authors, each oil from the *Amyris balsamifera* plant should be sold under its own name, for example, as amyris essential oil. Information about the scent notes and possible similarities to sandalwood oil can only be added as supplemental information. The English market is more honest in this regard: the *Amyris balsamifera* plant only appeared in the description on one English page. In contrast, the English market has the most oils from other sandalwood species compared with the German and Polish markets: *Santalum spicatum* (10%), *Santalum paniculatum* (4%) and *Santalum australedonicum* (2%).

*Santalum spicatum* comes mainly from southwestern Australia. This species is similar to *Santalum album* morphologically and physiologically. In contrast to white sandalwood, which is very expensive, sandalwood from Australia could have been used on a considerably larger scale in the nineteenth century due to its abundant occurrence in the natural environment. The oil obtained from *Santalum spicatum* differs from real *Santalum album* sandalwood oil, but it gained great popularity in perfumery and aromatherapy at the end of the twentieth century [24]. The essential oil from the *Santalum spicatum* tree differs in composition from the oil from *Santalum album*. The most important difference is the content of *α*-santalol and *β*-santalol, which in *Santalum spicatum* is significantly lower. The oil obtained from *S. spicatum* contains several other substances that increase the fragrance intensity, including farnesol, which occurs in trace amounts in white sandalwood [24]. Due to the differences in composition, *Santalum spicatum* oil has a slightly different effect than *Santalum album* oil. The component E, E-farnesol, which is unique to this particular oil, additionally has a sedative effect and can reduce the reaction to mental stimuli, for example, those caused by stimulant consumption. The same ingredient also enhances the effects of other disinfectants. Another ingredient not found in sandalwood oil is epi-alpha-bisabolol, which has an additional anti-inflammatory effect. The beneficial effect of *Santalum spicatum* oil in preventing the development of atherosclerosis has also been shown [24]. Australian sandalwood oil is considered less safe to use than *Santalum album* oil due to the high content of farnesol, which is considered a contact allergen [13]. The safety of white sandalwood oil was confirmed by a study in which only 0.1–2.4% of respondents showed hypersensitivity [7].

*Santalum paniculatum* is endemic only to Hawaii's largest island, the “Big Island.” This tree is the only Hawaiian sandalwood species currently commercially bred but in very limited quantities. Hawaiian sandalwood oil is rare and only sporadically available; it is characterized by a low content of *Z β*-santalol and high contents of *α*-santalum, E-*β*-santalol, and spirosantalol [35].

*Santalum austrocaledonicum* is less important in modern aromatherapy due to the small-scale production of the essential oil. This species of sandalwood can be found only in New Caledonia and the Republic of Vanuatu [4]. *Santalum austrocaledonicum* oil met the content of *α*- and *β*-santalol specified by ISO standards for sandalwood oil in only 19% of the studied trees from two islands of the Republic of Vanuatu [36].

The distinguishing feature of the English Internet market is the relatively frequent (24%) cases of *Santalum* essential oils dissolved in vegetable oils in the form of a ready-to-use product, which is not found on the German market at all and rarely found on the Polish market. Such a mixture often includes other essential oils or ingredients, such as vitamin E. This type of product is usually sold in larger packages in plastic bottles of various colors and shapes, while most essential oils are usually sold in 5, 10, and 15 mL dark glass bottles. In addition, the composition of such blends is described in detail, and it is difficult to mistake them for sandalwood essential oil from *Santalum album*, although the name “Sandalwood Essential Oil” sometimes appears in the photo, which should not happen.

A separate problem is the complete lack of the botanical name of the oil-bearing plant, both in the product photo and the description. Generally, German websites with poor descriptions (26% of websites) lead in this regard followed by Polish websites (18% of websites). Perhaps producers sell good-quality oil but with incorrect descriptions, but considering the overall view of the sandalwood oil market and the level of irregularities, the authors do not recommend buying sandalwood oil from such websites because one cannot tell what one will receive.

Unfortunately, not all websites that have listed the botanical name of the oil plant *Santalum album* can be trusted due to the price of the product. Good-quality sandalwood oil cannot be too inexpensive. *Santalum album* was a manifestation of the economy of plunder and excessive exploitation of natural resources for a long time. The most significant amount of oil is obtained from plants more than 30 years old. There was a deficiency of sandalwood oil on the market, which increased its price [2, 9, 10]. Polish companies selling real sandalwood oil checked by the chromatography method offered their product a minimum price of PLN 21 per 1 mL of oil, and the average price for oils considered by authors as good quality was PLN 28.19 per 1 mL. At the same time, on one of the seemingly reliable Polish websites with a full description containing the botanical name of *Santalum album*, one could buy sandalwood oil for an almost tenfold lower cost, notably, for PLN 2.64 per 1 mL. The authors considered this price an attempt to fraud and added the price criterion to the selection of good-quality products. When analyzing the prices of oils with the inscribed botanical name *Santalum album*, regardless of the language, their stratification can be observed. There are expensive and inexpensive products, and there are only a few products with an intermediate price, so the limit of PLN 21 adopted on the Polish market was also used on the German and English markets. The average prices for good-quality oils on these two markets were PLN 45.35 and PLN 50.79 per mL of product, respectively. The discrepancy in the average price of good-quality products depends on the economic conditions in a region, and the analysis of this issue is beyond the scope of this paper. However, to observe economic inequalities in the described markets, it is worth quoting the average annual salary: Poland = $ 31,970; Germany = $ 53,638; United States = $ 65,836 [37]. Perhaps the annual salary affects the percentage of high-quality sandalwood oils available online, which is 8% on Polish websites, 32% on German websites, and 38% on English websites.

The chance of buying good-quality sandalwood oil on Polish websites is also low because the real oil is not observed until the 10th position on displayed pages on Google, and it is difficult to imagine that an average buyer would visit 10 pages in search of sandalwood oil! The German and English markets are notably better in this respect, and good-quality sandalwood oils can be found on the first three websites.

The authors are aware of several limitations to this study and factors that might have influenced the results.

A significant factor determining the results of the work is the sandalwood oil quality criterion: the presence of the botanical name *Santalum album* in the product image and/or on the website and an item price of not less than PLN 21 per mL. In both cases, the results could have been distorted. Some of the oils without the botanical name specified may have actually come from *Santalum album*, but the distributor did not make an effort to correctly describe the product. The minimum limit of PLN 21/mL was obligatory based on prior knowledge regarding sandalwood oil on the Polish market and the shaping of prices, but perhaps a properly described oil for PLN 17/mL would also turn out to be of good quality after chromatographic examination. For the German and English markets, problems of a different type could arise; the threshold of PLN 21 may have been too low, and good products may have been considered to be of poor quality. There is also the issue of deliberate falsification of the oil, when sellers add less expensive cedar oil but leave a reliable price and the botanical name *Santalum album* [38].

Another important issue is the method of website searching. The authors made efforts to render this process as objective as possible by, for example, changing the location when changing the search language. However, the working algorithms of Google searches mean that the search history affects subsequent searches; for example, the order of pages might be related to previous searches for various cosmetic products. Having different people perform similar searches on different computers could also lead to differing results.

The most reliable method to assess the quality of sandalwood oil is chromatography. Considering the cost of conducting such research, the authors and online buyers had to rely only on information from sellers and their own knowledge. Despite the limitations of this work and possible inaccuracies in the classification of oils as good or questionable, the observations obtained indicate major shortcomings in the sale of sandalwood oil via the Internet, with the worst results being obtained on Polish websites, convincing the authors to publicize the results.

In Poland, sandalwood oil plays a significant role mainly in perfumery, in which its aromatic qualities are important [32]. It can be assumed that, for some consumers, essential oils are only supposed to perform the aroma function, and other aromatherapeutic properties are not as important. Nevertheless, it would be fairer to describe the product in a way in which the potential buyer, even without knowing the botanical name of the oil-bearing plant, would know whether he or she was buying a good-quality product or a less expensive alternative only with similar aromatic qualities. People who buy sandalwood oil for cosmetic or aromatherapy treatments must be particularly careful when buying online and should under no circumstances be guided by the low price.

## 6. Conclusions

Good-quality sandalwood oils derived from the *Santalum album* plant at a price equal to or greater than the chromatographically tested items amounted to a negligible percentage of products sold online. Without knowing the botanical name of the essential oil plant and the price range of unadulterated sandalwood oil, the likelihood of buying a reliable product is low on all of the analyzed websites, with the lowest probability being observed on Polish sites.

## Figures and Tables

**Table 1:** Good-quality oil (gray area) or the reason for excluding the oil as good quality in the first, second, and third positions from the top on the list of searched websites in Polish, German, and English.

Website position	Website language		
	Polish	German	English
1	Too low price	Good-quality oil	No botanical name
2	No botanical name	*Amyris balsamifera*	Good-quality oil
3	*Amyris balsamifera*	Good-quality oil	Too low price

## Data Availability

Data used in this paper are available upon request from the first author.
